# Clinical experience of reoperative right ventricular outflow tract reconstruction with valved conduits: risk factors for conduit failure in long-term follow-up

**DOI:** 10.1007/s10561-023-10088-y

**Published:** 2023-04-21

**Authors:** Mariia Havova, Roman Gebauer, Petra Antonova, Jaroslav Spatenka, Jan Burkert, Ondrej Fabian, Martin Modrak, Vilem Rohn

**Affiliations:** 1 Department of Cardiovascular Surgery, 2nd Faculty of Medicine, Charles University in Prague and Motol University Hospital, V Uvalu 84, 15006 Prague 5, Czech Republic; 2 Children’s Heart Centre, 2nd Faculty of Medicine, Charles University in Prague and Motol University Hospital, Prague, Czech Republic; 3 Department of Transplantation and Tissue Bank, National Allograft Heart Valve Bank, Motol University Hospital, Prague, Czech Republic; 4 Department of Bioinformatics, 2nd Faculty of Medicine, Charles University, Prague, Czech Republic; 5 Clinical and Transplant Pathology Centre, Institute for Clinical and Experimental Medicine, Videnska 1958/9, 140 21 Prague 4, Czech Republic; 6 Department of Pathology and Molecular Medicine, 3rd Faculty of Medicine, Charles University and Thomayer Hospital, Videnska 800, 140 59 Prague 4, Czech Republic

**Keywords:** Right ventricular outflow tract, Reoperation, Allograft, Valved conduit, Xenograft, Conduit failure

## Abstract

Reconstruction of right ventricular outflow tract in patients with congenital heart disease in various age groups remains a controversial issue. Currently, a little is known about the fate of secondary and subsequent conduit. The aim of the study was to determine risk factors of conduit failure, evaluate long-term conduit survival, find out which type of conduit should be preferred in case of reoperations. We performed a retrospective analysis of a total of 249 records of valved conduit secondary and subsequent replacement in right ventricular outflow tract in 197 patients. Median follow-up was 5.7 years. The study endpoints were defined as conduit explants; balloon dilatation of the graft (excluding balloon dilatation of left/right pulmonary artery), transcatheter pulmonary valve implantation; heart transplantation or death of the patient. There were total of 21 deaths (11% mortality) among 197 patients during the follow-up, 2 patients underwent heart transplant, in 23 implanted conduits pulmonary angioplasty or/including transcatheter pulmonary valve implantation was afterwards performed due to graft failure, conduit had to be explanted in 46 cases. After 28 years follow-up, freedom from graft failure after 5 years was 77%, 48% after 10 years and 21% after 15 years. Reoperative right ventricular outflow tract reconstruction demonstrates good mid-term and acceptable long-term outcomes regardless of the type of conduit implanted. Worse long-term graft survival of secondary and further conduits is associated with younger age of the recipient at implantation, small size of the conduit, younger age of donor and male donor in case of allograft implantation.

## Introduction

The possibilities of right ventricular outflow tract (RVOT) reconstruction in patients with congenital heart disease include allografts, xenografts, mechanical valved conduits and surgical valve reconstructions using pericardial patches or polytetrafluorethylene membranes (Brown et al. 2007; Brown 2018). As the "gold standard" for reconstruction of the RVOT is considered to be the use of allograft (Warrell et al. 2016). Nevertheless, a tendency toward allograft degeneration over the years is still apparent (van de Woestijne et al. 2011) and it is generally known that the future reoperation will most likely be required. Despite the improved methods of allograft preservation, a durable cardiovascular allograft has not yet been found—all patients develop early or late allograft failure or dysfunction (Vogt et al. 1999). Also, because of the allograft unavailability and problems with early allograft preservation, the use of porcine valve conduits was favored through the early 1980s (Koirala et al. 2002). Late complications however, led to decreased use of porcine valve conduits (Jonas et al. 1985). 

Long-term results of RVOT reconstruction with valved conduits in reoperations have been scarcely reported thus far and the conduit of choice after first conduit explanation still remains a questionable issue. In our study a retrospective evaluation of reoperative conduit RVOT reconstruction was reviewed to determine risk factors of conduit failure in order to propose potential ways to improve the long-term results and find out which conduit should be preferred in case of reintervention.

## Materials and methods

The study was approved by the local research ethic committee—Reference No.: EK-930/18 (15.8.2018). Patient consent was not required.

### Patient characteristics

The retrospective study included all children and adult patients, who underwent second and further RVOT reconstruction with a valved conduit between October 1988 and October 2016 in our center. In this period, a total of 249 reimplantations of both types of conduits were performed in 197 patients (age 13 days to 42 years). Median follow-up was 5.7 years with the earliest conduit explant 133 days after implantation. Among the whole group of reimplantations 191 allografts were used for the RVOT reconstruction (127 pulmonary, 57 aortic, in 7 cases the data regarding the type of the allograft conduit were not available due to their older origin). In 53 cases xenografts were used (in case of secondary and subsequent implantation, only Hancock Porcine-valved Dacron conduits, DuPont, Wilmington, DE) and remained 5 interventions with unknown conduits (due to the older origin of data). The conduit size was split into tertiles (9–21 mm, 22–25 mm, 26–31 mm) and in half (9–24 mm, 25–31 mm). The patient population consisted of 106 males (54%) and 91 females (46%). Donor age was split into tertiles (at 22 years and 36 years) and roughly in half (29 years). From the 249 performed conduit reimplantations 197 were secondary operations, 43 were tertiary operations and 9 reimplantations were performed as quaternary. The patient initial diagnoses included tetralogy of Fallot (TOF)—38 cases, transposition complexes (TGA, cTGA)—30 cases, pulmonary atresia and stenosis (PA and PS)—62 cases, double outlet right ventricle (DORV)—28 cases, truncus arteriosus (TA)—73 cases and the group of patients, who underwent Ross/Ross–Konno procedure due to congenital heart disease of aortic valve (aortic stenosis (AS) or aortic insufficiency (AI)—18 cases. Table 1 provides the patients initial diagnosis with stratified main properties for all conduits and Table 2 represents the summary of allograft—specific properties stratified by diagnosis.

**Table 1:** Summary of main properties of all the conduits stratified by diagnosis

	CTGA	DORV	PA	PTA	ROSS	TGA	TOF
N interventions	4	28	62	73	18	26	38
Recipient male	3 (75%)	18 (64%)	25 (40%)	32 (44%)	15 (83%)	20 (77%)	20 (53%)
Age at intervention (mean, IQR)	18 (14–21)	13 (8–16)	14 (6–20)	9 (4–13)	12 (6–16)	16 (12–19)	15 (8–21)
Age at intervention (range)	12–28 years	2–27 years	242 days–42 years	13 days–24 years	119 days–29 years	2–36 years	1–33 years
Conduit size (mm, mean, IQR)	24 (22–26)	24 (22–26)	22 (18–26)	21 (18–25)	23 (21–26)	24 (22–26)	23 (21–26)
Cross clamp used	0 (0%)	10 (40%)	13 (29%)	33 (46%)	4 (27%)	4 (19%)	7 (21%)
Cross clamp time (mean, IQR)	N/A	85 (74–98)	83 (51–117)	101 (62–128)	98 (61–116)	65 (51–89)	57 (32–85)
Cross clamp information missing	0 (0%)	3 (11%)	17 (27%)	1 (1%)	3 (17%)	5 (19%)	5–13%
Temperature (mean, IQR)	33 (33–34)	31 (28–34)	31 (30–34)	30 (28–33)	31 (28–34)	27 (20–33)	27 (28–34)
Temperature missing	0	3	16	1	4	5	3
Time of bypass [min] (mean, IQR)	167 (126–192)	195 (120–255)	188 (134–230)	184 (116–224)	155 (93–202)	171 (118–201)	170 (116–207)
ECC	4 (100%)	28 (100%)	61 (100%)	73 (100%)	18 (100%)	25 (100%)	36 (95%)

**Table 2:** Summary of allograft—specific properties stratified by diagnosis

	CTGA	DORV	PA	PTA	ROSS	TGA	TOF
N allografts	2	20	44	58	15	22	30
Pulmonary allograft	0 (0%)	14 (70%)	32 (73%)	38 (66%)	11 (73%)	10 (45%)	22 (73%)
Aortic allograft	2 (100%)	6 (30%)	11 (25%)	19 (33%)	4 (27%)	10 (5%)	5 (17%)
Allograft cryopreserved	2 (100%)	19 (95%)	41 (93%)	53 (91%)	15 (100%)	17 (77%)	26 (87%)
Allograft storage unknown	0 (0%)	0 (0%)	1 (2%)	2 (3%)	0 (0%)	2 (9%)	3 (10%)
Time from harvest to preservation (days, mean, IQR)	20 (18–22)	22 (16–26)	21 (17–27)	21 (14–27)	26 (13–26)	23 (19–27)	22 (18–28)
Time from harvest to preservation missing	0 (0%)	6 (32%)	13 (32%)	13 (25%)	1 (7%)	8 (47%)	7 (27%)
Donor male	2 (100%)	12 (60%)	21 (48%)	32 (55%)	7 (47%)	11 (50%)	13 (43%)
Donor sex missing	0 (0%)	0 (0%)	1 (2%)	2 (3%)	0 (0%)	2 (9%)	4 (13%)
Donor age (years, mean, IQR)	34 (31–37)	32 (20–44)	33 (20–46)	26 (16–35)	31 (20–40)	32 (22–44)	31 (19–42)
Donor age missing	0 (0%)	0 (0%)	1 (2%)	2 (3%)	0 (0%)	2 (9%)	4 (13%)
ABO match	1 (50%)	8 (42%)	21 (50%)	34 (62%)	7 (47%)	9 (47%)	16 (67%)
ABO missing	0 (0%)	1 (5%)	2 (5%)	3 (5%)	0 (0%)	3 (14%)	6 (20%)

### Allograft characteristics

All the allografts were harvested, processed, cryopreserved and allocated by Department of Transplantation and Tissue Bank (Ministry of Health of Czech Republic Code: STB85), National Allograft Heart Valve Bank, Motol University Hospital, Prague. Hearts for the preparation of valve allografts were obtained in accordance with Czech legislation: the "Transplantation Act" (Act No. 285/2002 Sb.) and the "Act of Human Cells and Tissues" (Act No. 296/2008 Sb.) (Hlubocký et al. 2011). The age limit for the donor was set from matured newborn up to 65 years. Processing and storage of the allografts as well as allograft thawing was performed according to standard protocols. Shelf life of cryopreserved allografts was arbitrarily set on 5 years (Burkert et al. 2008, 2021; Fiala et al. 2019).

In the case of small children, except for CT scan measuring, we used nomograms (e.g. patient’s body surface area) to assess the normalized diameter of the allograft, in some cases allograft was reduced in size by being bicuspidalized.

### Operative techniques

The surgical procedures were performed through median sternotomy, on beating hearts, with the use of standard cardiopulmonary bypass with mild hypothermia or normothermia. If associated intracardiac procedures were required, the aorta was cross-clamped and myocardial protection was performed using blood or crystalloid cardioplegia. The allograft was sewn between the right ventricle and pulmonary artery, using the interposition technique. The anastomoses were made with a running polypropylene suture. The mean time of cardiopulmonary bypass was 181 min, mean time of aortic cross-clamping (if used) was 89 min.

### Data collection

Follow-up data of patients were retrospectively collected from the database and hospital records. The day of second and further implantation of each graft was considered the starting point of graft survival. Clinical follow-up was performed by pediatric (patients < 19 years) and adult cardiologist experienced with congenital heart diseases (patients > 19 years) at our center, and follow-up data were subsequently obtained from medical records of the hospital. Clinical variables assessed as possible negative predictors included: conduit type, patients age, patients’ sex, initial diagnoses, the number of reintervention and type of surgery (Ross/non-Ross groups). In case of allograft implantation possible examined risk factors were the following: allograft type, diameter, method of preservation, time from harvesting to preservation and time of preservation itself, donor age, donor sex, AB0 matching. The study endpoint was conduit failure, defined as conduit explanations; conduit-related intervention without explants: balloon dilatation of the conduit (excluding balloon dilatation of left/right pulmonary artery distal to the conduit), transcatheter pulmonary valve implantation (TPVI); heart transplantation and death of the patient. Exclusion criterium was infective endocarditis on pulmonary valve before the current operation.

### Statistical methods

To assess association between individual variables and outcomes we used the Cox proportional hazards model as implemented in the R package survival (Therneau and Grambsch 2000). For each comparison, we run two analyses: an unadjusted model where we compare a single-covariate model to a model without any predictor and an adjusted model where we include four associations found in previous work (age at intervention, conduit size, type, and primary diagnosis) and compare model with those four predictors and the covariate of interest to a model with just the four predictors. For the comparisons we use the likelihood ratio test. All p-values reported in the main text were adjusted for multiple comparisons using the Benjamini–Hochberg method (Hochberg and Benjamini 1990) for controlling false discovery rate at 5%. Survival curves and their 95% confidence intervals were visualized using the R package survminer (Kassambara et al. 2017).

## Results

**Fig. 1:**
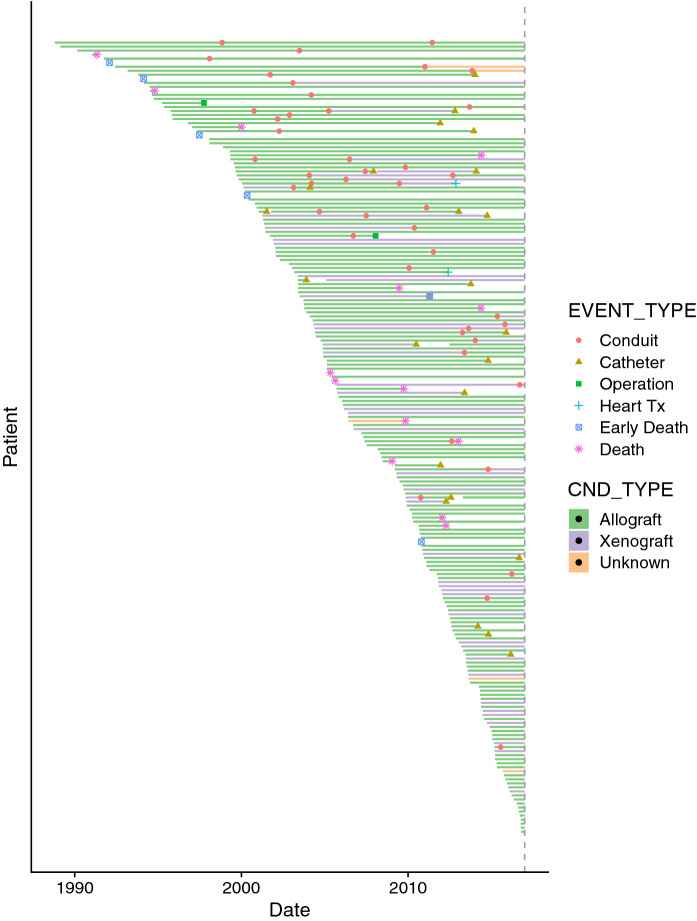
A plot of all conduits and events, including early mortality. Each row is a single patient. The vertical dashed line represents the end of data collection

There were total of 21 deaths (11% mortality) among 197 patients during the follow-up. Two patients underwent heart transplant afterwards. In 23 implanted conduits pulmonary angioplasty or/including transcatheter pulmonary valve implantation was afterwards performed due to conduit failure. Allograft had to be explanted in 52 cases. 5-year freedom from graft failure in all conduits was 77% (75% in allografts and 88% in Hancock Porcine-valved Dacron conduits), 10-year freedom from graft failure was 48% (50% in allografts and 44% in Hancock Porcine-valved Dacron conduits), 15-year freedom from graft failure was 21% with 23% in allografts and 11% in Hancock Porcine-valved Dacron conduits respectively. Four conduits are in place 20 years after implantation. Earliest allograft was replaced in 13 days after the day of implantation of primary conduit. Infective endocarditis was detected in eight cases. Early mortality (30 day mortality after surgery and perioperative mortality) assessed 3% (6 patients and none of them was conduit related). Representation of conduits and events is introduced in Fig. 1.

### Risk factors for conduit failure

Comparing the type of conduit (allograft vs. Hancock Porcine-valved Dacron conduit) in reintervention, the unadjusted model does not show statistically significant difference survival and is consistent with both large increase and decrease in survival (p = 0.84, likelihood ratio test, the 95% CI for hazard ratio is (0.64–1.95). In the adjusted model we also do not see a significant difference for conduit type—allograft vs. Hancock Porcine-valved Dacron conduit (p = 0.09, likelihood ratio test) and the associated confidence for hazard ratio of Hancock Porcine-valved Dacron conduit over allograft is (0.26–0.99), so under the adjusted model, the data are mostly inconsistent with increased hazard associated with Hancock Porcine-valved Dacron conduits. Comparing pulmonary and aortic allograft separately in unadjusted survival model provides weak evidence that included subtype creates an association between graft type and conduit survival (p = 0.06, likelihood ratio test), the hazard ratio for pulmonary allografts vs. aortic allografts is (0.29–0.83) and for Hancock Porcine-valved Dacron conduits vs. aortic allografts is (0.41–1.41). Computing the adjusted comparison, gives us a similar significance level (p = 0.09, likelihood ratio test) and similar hazard ratios for pulmonary allografts vs. aortic allografts (0.37–1.2) and for Hancock Porcine-valved Dacron conduits vs. aortic allografts is (0.22–0.86) so substantial uncertainty about the association remains. Cryopreserved allografts were used more often in the included diagnoses (173 cryopreserved, 10 fresh, 8 of unknown origin), which also gives non-significant comparison with very wide uncertainty (p = 0.34 in unadjusted model with hazard ratio 0.87–10.01).

Comparing subgroups of secondary, tertiary, and quaternary implantations in the adjusted model, tertiary conduits are unlikely to be associated with substantially lower hazard than secondary conduits, although we cannot usefully constrain any potentially increased hazard and the data are consistent with no difference—the 95% CI for hazard ratio is (1.0–3.65). In the unadjusted model the confidence intervals are too wide for any useful conclusions.

**Fig. 2:**
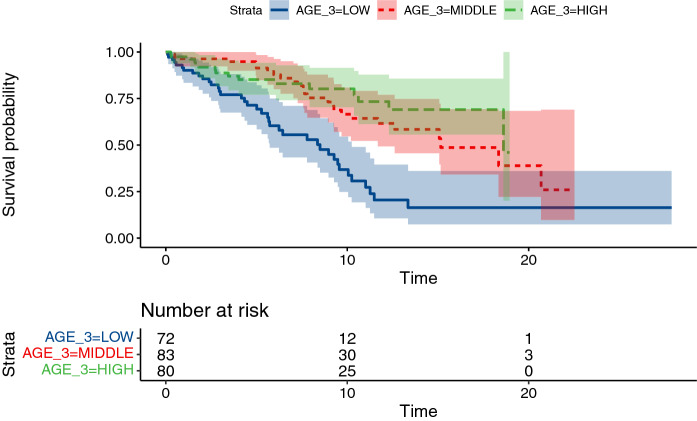
Kaplan–Meier curves for conduit failure stratified by tertiles of recipient age (0–8 years, 8–16 years, 16–42 years)

There was a robust negative association of older age at intervention with longer graft survival. This relationship is significant under all tested model variants (all p < 0.02, likelihood ratio test). The 95% CI for hazard ratio between lowest and highest tertile of age at intervention is 0.16–0.52 (Fig. 2) Mean recipients age at implantation of the conduits was 13 years, with a range of 13 days after birth to 42 years.

**Fig. 3:**
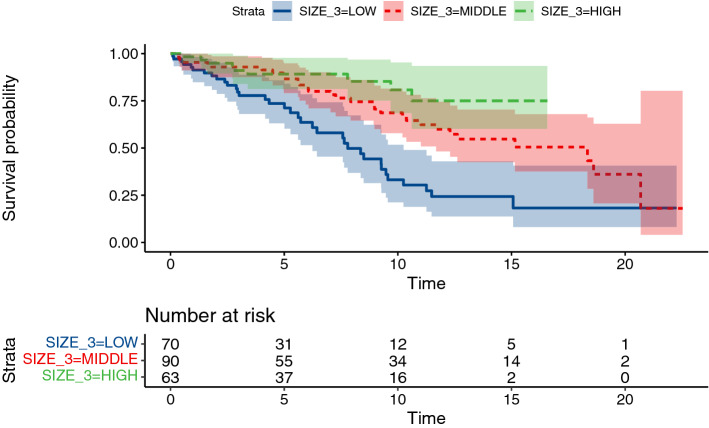
Kaplan–Meier curves for conduit failure stratified by tertiles of conduit size

For the unadjusted model, we tested both splitting the conduit size roughly into tertiles (9–21 mm, 22–25 mm, 26–31 mm, see Fig. 3) and roughly in half (9–24 mm, 25–31 mm). In unadjusted models, larger conduit size is associated with longer graft survival (p < 0.001, likelihood ratio test). In the adjusted model, the resulting 95% CI for hazard ratio of conduits 10 mm apart in size (0.17–1.06) does not allow for strong conclusions.

**Fig. 4:**
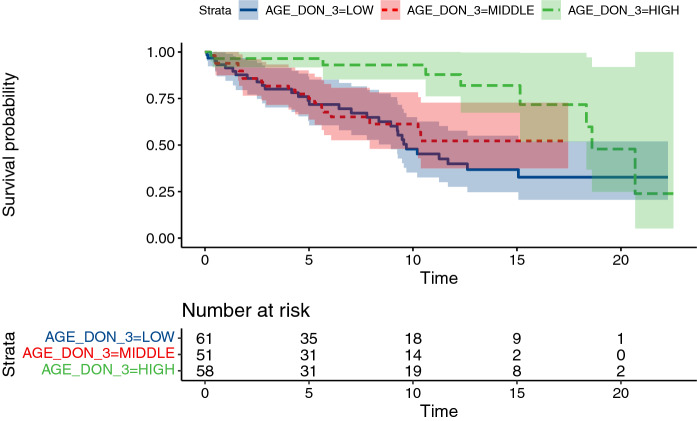
Kaplan–Meier curves for conduit failure stratified by tertiles of donor age

In unadjusted models, higher donor age is associated with longer conduit survival (p < 0.001, likelihood ratio test), however the association can be potentially ascribed to the correlation between donor age and age at intervention or conduit size. In the adjusted model, the resulting 95% CI for hazard ratio of donors one year apart (0.96–1.01) rules out strong associations (Fig. 4).

**Fig. 5:**
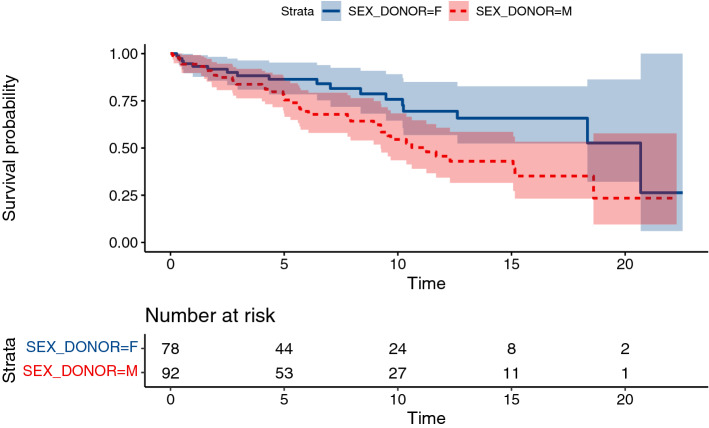
Kaplan–Meier curves for conduit failure stratified by donor sex

**Fig. 6:**
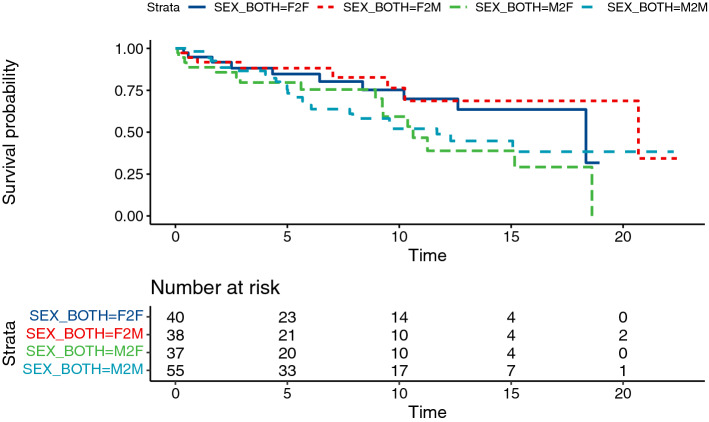
Kaplan–Meier curves for conduit failure stratified by donor sex match/mismatch. *F2F* a female donor and female recipient, *F2M* a female donor and male recipient, *M2F* a male donor and female recipient, *M2M* a male donor and male recipient

Unexpectedly, conduits from male donors are associated with shorter graft survival in both unadjusted and adjusted models (p = 0.03, likelihood ratio test, 95% CI for hazard ratio 1.2–3.93) (Fig. 5). Furthermore, we tested a sex match/mismatch between donor and recipient, where we cannot make strong conclusions (p = 0.84, hazard ratio 0.62–1.78). Additionally, we tested all four combinations of donor and recipient sex, where a possible difference cannot be proven nor rejected (Fig. 6).

## Discussion

For the first time, RVOT allograft implantation technique was described by Ross and Somerville (1966). This has resulted in ever increasing application of allografts, which were further widely used for the correction of complex congenital heart defects (Cleveland et al. 1992). Cryopreservation solved the storage and availability problems that plagued the early use of allografts (Albert et al. 1993). Extending of allograft expiration period over 5 years might improve the allograft availability (Fiala et al. 2019; Burkert et al. 2021). Nowadays, allograft heart valves became slowly conduits of choice in both primary operations and reoperations in the RVOT reconstruction. Benefits of their use generally include ease of implantation, better hemodynamics, improving right ventricle function after operation, their resistance to infection, decreased number of thromboembolic events, lack of need for anticoagulation and the possibility to use the branch of allograft as a patch for distal pulmonary artery stenoses.

RVOT reconstruction with an allograft conduit can be performed with good patient survival, acceptable long-term allograft durability, and good perceived quality of life. In the study from Brown et al. (2005) reported a freedom from allograft failure of 60% at 5 years and 43% at 15 years, Niwaya et al. (1999) reported a freedom from allograft failure of 82% at 8 years, Stark et al. (1998) described 58% and 31% freedom from conduit replacement at 10 and 15 years, respectively. Bielefeld et al. (2001) reported freedom from early death, valve-related late death, surgical or catheter intervention without replacement, or allograft explant in total 35 patients who underwent allograft explant at 5, 10, and 12.4 years postoperatively was 89% (95% CL 79–100%), 76% (95% CL 49–100%), and 76% (95% CL 49–100%), respectively. 

However, recent studies have demonstrated disappointing mid- and long-term results when used in neonates, infants, and children (da Costa et al. 2017). Our data showed the following results: 5-year freedom from graft failure in all conduits was 77% (75% in allografts and 88% in Hancock Porcine-valved Dacron conduits), 10-year freedom from graft failure was 48% (50% in allografts and 44% in Hancock Porcine-valved Dacron conduits), 15-year freedom from graft failure was 21% with 23% in allografts and 11% in Hancock Porcine-valved Dacron conduits respectively, which are different from the data reported by Bielefeld and coworkers. Comparisons to studies that describe allograft or xenograft survival in pediatric populations are difficult because these conduits are smaller and mostly placed in a nonorthotopic position (Niwaya et al. 1999). Kalfa et al. (2012) report better long-term results for patients who underwent Ross or Ross–Kono procedures than patients with other congenital hearts defects. This may be because the allograft is implanted in an orthotopic position, which reduces turbulence in the conduit and increases its durability. In addition, it is possible to oversize the allograft to a much greater degree than in other operations. Although in our study comparison of allograft survival in patients from Ross and non-Ross did not result as statistically significant—most likely because of the small number of patients in Ross group, where the incidence of secondary and subsequent replacements is rare—which can also confirm better long-term survival of the allograft in the following group.

Cumulative mortality in our study was 11% (21 patients) in 197 recipients of secondary and further conduits implanted to the RVOT. Their main preoperative diagnoses were truncus arteriosus (TA)—6 patients, pulmonary atresia and stenosis (PA and PS)—6 patients, tetralogy of Fallot (TOF)—4 patients, transposition complexes (TGA, cTGA)—4 patients, double outlet right ventricle (DORV)—2 patients and 1 patient, who underwent Ross procedure. We observed early mortality (defined as perioperative and 30 day mortality after surgery) of 3%. Mortality is probably mainly associated with early surgical era and patients’ primary diagnosis (TA and PA and PS). Those data are consistent with the data referred by Rodefeld and coworkers, where the diagnosis of truncus arteriosus (p = 0.001) and surgery before 1992 (p = 0.05) remained significant by multivariate analysis (Rodefeld et al. 2008).

Negative predictors of accelerated allograft degeneration according to the previous studies also include small size of allograft, young age of recipient, aortic type of allograft and duration of warm ischemia (Bielefeld et al. 2001; Tweddell et al. 2000; Baskett et al. 1996). Our data were consistent regarding the conduit size and age at intervention. In our results, in case of small allograft size we should however note that there was a strong correlation between conduit size and age at intervention. There also was an association of longer preservation with longer conduit survival (p = 0.02). However, time of preservation is positively correlated with age at intervention and the same comparison in the adjusted model is not significant. (p = 0.7) In our study there was some mild evidence for additional differences due to graft type, however, just as in case of preservation time, those results were potentially false positive and do not result in significant differences after multiple testing correction.

Although in current research, we did not study reoperation itself as the risk factor for conduit failure, comparing the primary with any subsequent conduit does not provide significant difference (with p = 0.57, likelihood ratio test). According to Bielefeld et al. graft survival for replacement conduits turned out to be similar to primary allografts—although not statistically significant, actuarial event-free curves for primary and reoperative allografts demonstrated improved graft survival in the replacement allograft group (Bielefeld et al. 2001). Meyns et al. (2005) also states that the second allograft performs as well as the first where Kaplan–Meier allograft survival curve for the first and the second allografts implanted in the subgroup of patients, who actually received two homografts, illustrates that the survival of the second graft is slightly better than the first graft. Clearly, in this setting, one has to remind that the patients are older when they receive their second graft. The results are also consistent with Lewis et al., which demonstrated no evidence for a shorter life span of the second graft (Lewis et al. 2023). 

In our cohort, conduits from female donors tend to have lower failure risk. This is unexpected in light of previous reports of Kalfa et al., which showed that female sex of the donor allograft significantly alter the outcomes of the allograft in the multivariate analysis. According to their study, choosing allografts coming from a male donor older than 30 years, if available, is recommended to reconstruct the right ventricular outflow tract (Kalfa et al. 2011). However, in our study, further testing the donor/recipient sex match/mismatch did not result in statistically significant difference. So, the influence of donor sex on graft survival is still not clear and the reasons underlying should be confirmed in the further research focusing on donor/recipient sex-mismatch in larger series.

The comparisons for initial diagnosis, type of surgery (Ross vs. non-Ross group), time from harvesting to preservation, recipient sex and ABO matching also yield inconclusive results (p = 0.87, 0.30, 0.84, 0.70, 0.98 respectively likelihood ratio test, the 95% CI for hazard ratios for ROSS vs. other diagnosis is (0.82–3.92) for conduits preserved a week apart is (0.87–1.32) for male sex is (0.51–1.38) and for ABO matching is (0.67–2.03) so in all cases the data are consistent with both substantial increases and decreases in survival across the various categories.

Allografts are generally limited in the smaller sizes (below 16 mm). So, allograft availability is an issue for the infant and neonates. Downsizing of adult allografts, to solve this problem, was introduced by some surgeons by removing one semilunar valve leaflet with a corresponding strip of the conduit. This converts the valve to a bicuspid arrangement, but this appears to function well in vitro tests and in patients (Koirala et al. 2002). In our cohort of secondary and subsequent allografts, we had only 4 bicuspidalized allografts. All of them were cryopreserved pulmonary allografts with primary patient’s diagnosis of truncus arteriosus. Earliest allograft was replaced in 9 months after the day of implantation, conduit failure for the remaining conduits was registered in 46, 52 and 57 months after implantation. This worse long-term survival can be probably associated with younger age of the recipient at implantation, small size of the conduit, which, according to our study, resulted as significant risk factors of conduit failure and primary diagnosis of truncus arteriosus, which was revealed to be one of the significant risk factors for need of replacement according to Rodefeld et al. (2008).

In the future, we can expect a further improvement in the fate of allografts by their decellularization. Use of decellularized allografts for RVOT reconstruction in children was associated with a low incidence of structural valve deterioration and conduit failure in some studies. Although these results still need to be confirmed in larger series and with longer follow-up, recent data suggest favorable outcomes, in the first decade after the operation (Cleveland et al. 1992).

### Study limitations

A limitation of the study is the retrospective design subjected to all limitations associated with this type of research. The study was limited by the lack of data at seven cases. In those cases, the complete data regarding the conduit were not available due to their older origin.

Our study focused on the endpoint of valve or conduit failure, we did not include dysfunction at various degrees, which did not lead to replacement, balloon dilatation of the implanted conduit, percutaneous valve implantation, heart transplantation or patient death.

For interpreting the unadjusted model results (where we include associations from the previous work—age at intervention, conduit size and type) we should note that there is substantial correlation between many of the predictors and thus the associations of any individual predictor with outcome cannot be safely interpreted as predictive beyond the sampling frame of study. This limitation is however slightly alleviated in the adjusted model, but there are many other correlations that are not handled by the adjustments.

## Conclusion

Reoperative RVOT reconstruction is possible, with good mid-term and acceptable long-term event-free graft survival and can be accomplished with reasonably low morbidity and mortality, although conduit failure and explant are inevitable. Our study showed that long-term survival between allografts and Hancock Porcine-valved Dacron conduits in the RVOT for secondary and subsequent conduits shows no statistically significant differences, which means that both types of conduits can be successfully used for the reoperative reconstruction of the RVOT. Among 249 cases of conduit reimplantation in patients undergoing the RVOT reconstruction with both types of conduits, worse long-term graft survival was associated with younger age of the recipient at implantation, small size of the conduit, younger age of donor and male donor in case of allograft implantation.

## Data Availability

The datasets generated during and/or analysed during the current study are available from the corresponding author on reasonable request.
